# The SET Domain Is Essential for Metnase Functions in Replication Restart and the 5’ End of SS-Overhang Cleavage

**DOI:** 10.1371/journal.pone.0139418

**Published:** 2015-10-05

**Authors:** Hyun-Suk Kim, Sung-Kyung Kim, Robert Hromas, Suk-Hee Lee

**Affiliations:** 1 Department of Biochemistry & Molecular Biology, Indiana University School of Medicine, Indianapolis, Indiana, United States of America; 2 Department of Medicine, University of Florida and Shands Health Care System, Gainesville, Florida, United States of America; 3 Indiana University Simon Cancer Center, Indianapolis, Indiana, United States of America; Saint Louis University, UNITED STATES

## Abstract

Metnase (also known as SETMAR) is a chimeric SET-transposase protein that plays essential role(s) in non-homologous end joining (NHEJ) repair and replication fork restart. Although the SET domain possesses histone H3 lysine 36 dimethylation (H3K36me2) activity associated with an improved association of early repair components for NHEJ, its role in replication restart is less clear. Here we show that the SET domain is necessary for the recovery from DNA damage at the replication forks following hydroxyurea (HU) treatment. Cells overexpressing the SET deletion mutant caused a delay in fork restart after HU release. Our *In vitro* study revealed that the SET domain but not the H3K36me2 activity is required for the 5’ end of ss-overhang cleavage with fork and non-fork DNA without affecting the Metnase-DNA interaction. Together, our results suggest that the Metnase SET domain has a positive role in restart of replication fork and the 5’ end of ss-overhang cleavage, providing a new insight into the functional interaction of the SET and the transposase domains.

## Introduction

Metnase is a SET [Su(var)3-9, Enhancer-of-zeste, Trithorax] and transposase chimeric protein with multiple functions in non-homologous end joining (NHEJ) repair, restart of stalled replication forks, DNA integration, and chromosomal decatenation [1–12]. Metnase enhances cell proliferation and cell survival after replication block induced by hydroxyurea (HU) and other DNA damaging agents [2, 5]. Overexpression of Metnase increased NHEJ repair, while it caused little change in recombination repair [6]. Similarly, cells treated with Metnase-siRNA showed a significant reduction in NHEJ repair activity *in vivo* [6]. A deletion of either SET or transposase domain abrogated Metnase’s function in DNA repair, indicating that both domains are required for this function [6, 13].

The transposase domain of Metnase contains the catalytic motif conserved among transposase and retroviral integrase families [14, 15]. It possesses most of the transposase activities, including the binding to terminal inverted repeat (TIR), the assembly of a paired end complex, cleavage of the 5'-end of the TIR element, and the promotion of integration at a TA target site [13, 16–20]. Unlike transposase, however, Metnase has a unique DNA endonuclease activity that mediates cleavage of duplex DNA in the absence of TIR sequence [20]. The catalytic motif is crucial for its DNA endonuclease activity as a point mutation at this motif (DDN → DDD/E) abolished its DNA cleavage activity [5, 20]. Cell extracts lacking Metnase poorly supported DNA end joining, but adding back of wt-Metnase not a mutant defective in endonuclease activity (D483A) markedly stimulated DNA end joining [1], suggesting that Metnase’s endonuclease activity is essential for promoting end joining. Metnase-mediated endonuclease activity preferentially acts on the ssDNA overhang of a DNA substrate [1, 5], which may play a crucial role in DNA end joining and replication restart [1, 5]. Interestingly, the catalytic domain of Metnase binds ssDNA but not dsDNA, whereas dsDNA binding activity resides in the helix-turn-helix DNA binding domain [5]. Substitution of Asn-610 with either Asp or Glu within the catalytic motif significantly reduces ssDNA binding activity [5], suggesting that the catalytic site of Metnase is directly in contact with the 5′-terminus for Metnase loading onto ss-overhang of DNA substrate.

The SET domain was initially identified as part of a conserved region in the *Drosophila* Trithorax protein and was subsequently identified in the *Drosophila* Su(var)3-9 and 'Enhancer of zeste' proteins, from which the acronym SET is derived. Although the methylation of histone residues is widely believed to be the central function of the SET domains, important aspects of this process, such as how SET proteins are recruited in the first place and how the histone modifications survive replication, are not understood. The SET domain of Metnase comprises pre-SET, SET, and post-SET domains. The Pre-SET domain contains a cysteine- and histidine-rich putative Zn^+2^ binding motif, and the SET domain has the conserved the histone lysine methyltransferase (HLMT) motif shared with other SET proteins in humans [3, 6]. A recent study showed that DSB damage induces dimethylation of histone H3 at lysine 36 (H3K36me2) in human cells [3, 6]. Chromatin immunoprecipitation (ChIP) and immunoblot analyses indicated that H3K36me2 is formed at DSB sites [3]. H3K36me2 is associated with chromatin opening [21–27], which may also be a part of its DSB localization via chromatin modulation [3, 21, 28]. Levels of DSB-induced H3K36me2 strongly correlate with Metnase expression and that the mutant lacking HLMT activity fails to generate H3K36me2, suggesting that Metnase is responsible for the induction of H3K36me2 at DSB site [3]. Metnase-mediated dimethylation of H3K36 at DNA damage site(s) perhaps enhances recruitment of Ku80 and perhaps other DNA repair factors to the damage site [3]. H3K36me2, once formed at DSB sites, may create docking sites for other repair proteins, recruiting them for DNA repair [3, 29]. In fact, mutations at known conserved SET domain amino acids significantly lowered DNA end joining [6], suggesting that Metnase’s HLMT activity plays a crucial role in early stage of DSB repair [29]. In this study we found that the Metnase SET domain is positively involved in the recovery from DNA damage at the replication fork and in the restart of stalled replication fork. The Metnase SET domain is essential for the 5’ end of ss-overhang cleavage with fork and non-fork DNA, which may be directly linked to Metnase function in replication restart.

## Materials and Methods

### Chemicals, DNA substrates, and antibodies

The following suppliers provided the listed items: [α- and γ-^32^P]-ATP (3000 Ci/mmol) from Perkin-Elmer (Boston, MA), DE81 filters from Whatman Bio System (Maidstone, England), and Bradford reagents and protein molecular weight markers were purchased from Bio-Rad (Hercules, CA). The oligonucleotides were obtained from the Integrated DNA Technologies (Coralville, IA). An anti-FLAG antibody was obtained from Sigma (St. Louis, MO). The oligonucleotides and the 5’-fluoresent labeled DNA were obtained from the Integrated DNA Technologies (Coralville, IA).

### Cloning and Purification of wt-Metnase and the mutants

Wt-Metnase was subcloned into pFLAG-CMV4 (Sigma) at HindIII and BamHI sites after PCR amplification with the forward primer (5’- CCC AAG CTT GGG ATG GCG GAG TTT AAG GAG-3’) and the reverse primer (5’-CGC GGA TCC GCG TTA ATC AAA ATA GGA ACC ATT ACA-3’). The SET deletion mutants were generated by PCR using specific primers: Δpre-SET (5’-CCC AAG CTT GGG GTG GTC CAG AAA GGT CTA-3’; 5’-CGC GGA TCC GCG TTA ATC AAA ATA GGA ACC ATT ACA-3’); Δall-SET (5’- CCC AAG CTT GGG ATG AAA ATG ATG TTA GAC-3’; 5’-CGC GGA TCC GCG TTA ATC AAA ATA GGA ACC ATT ACA-3’); ΔSET (5’- CCC AAG CTT GGG ATG GCG GAG TTT AAG GAG-3’; 5’-CCG GAA TTC CGG CAC TCT GTT TCT GCA GTG-3’; 5’-CCG GAA TTC CGG GAA AGG CTA GAT CAT GGG-3’; 5’-CGC GGA TCC GCG TTA ATC AAA ATA GGA ACC ATT ACA-3’); and Δpost-SET (5’- CCC AAG CTT GGG ATG GCG GAG TTT AAG GAG-3’; 5’-TGC TCT AGA GCA GTC TTC ACT GAC TGT TAG ATT AAG-3’; 5’-TGC TCT AGA GCA ATG AAA ATG ATG TTA GAC-3’; 5’-CGC GGA TCC GCG TTA ATC AAA ATA GGA ACC ATT ACA-3’). Wt-Metnase and the mutants were purified from HEK293 cells stably expressing flag-tagged Metnase as described [13, 20]. Flag-Metnase was detected in cell extracts by Western blotting using a monoclonal antibody (Sigma M1) as described previously [13, 20]. Cells (1.6 x 10^8^) overexpressing wt-Metnase or the mutant were suspended in 20 ml of extraction buffer (TEGDN; 50 mM Tris-HCl pH 7.5, 1 mM EDTA, 10% glycerol, 5 mM DTT, 1.0% Nonidet-P40, and mammalian protease inhibitor cocktails containing 0.2M NaCl), and centrifuged (100,000 x g) for 30 min. The supernatant (S100 fraction) was passed through a Whatman filter paper and was incubated at 4°C for 60 min with anti-FLAG M2 affinity gel (Sigma) that had been pre-equilibrated with TEGDN buffer containing 0.2 M NaCl. The beads were washed three times with TEGDN-2.0 M NaCl buffer prior to elution of the protein with TEGDN-0.2 M NaCl containing FLAG peptide (500 μg/ml). The eluant was diluted with 10 volumes of TEGDN buffer, and loaded onto a heparin-Sepharose 6 Fast Flow column (Amersham Biosciences) pre-equilibrated with TEGDN buffer. After washing the column, Metnase was fractionated using a linear gradient (0 to 2.0 M NaCl) of TEGDN buffer. The eluted protein was dialyzed against TEGDN buffer containing 50 mM NaCl and stored at -80°C.

*SDS-PAGE and Western blot*—Protein was analyzed by 10% SDS-polyacrylamide gel electrophoresis (SDS-PAGE). For Western blot analysis, proteins were transferred to polyvinylidene difluoride (PVDF) membrane, probed with an anti-FLAG antibody (monoclonal mouse IgG, Sigma) followed by horseradish peroxidase-conjugated secondary antibody. Protein was visualized by using the ECL system (Amersham Biosciences).

### Preparation of ^32^P-labeled DNA substrates

For 5’-^32^P labeling of DNA, 40 pmol of indicated ssDNA was incubated with 30 units of polynucleotide kinase (PNK; Affymetrics, Cleveland, OH) in the presence of γ-^32^P-ATP according to the manufacturer’s protocol. The ^32^P-ssDNA was then annealed to non-labeled DNAs to prepare various DNA substrates for DNA cleavage assay.

### DNA cleavage assay in vitro

For preparation of DNA substrates used in DNA cleavage assays, oligonucleotides were mixed and annealed together for 5’-flap DNA (5’-^32^P-labeled 5’-CGATACTGAGCGTCACGGACTCTGCCTCAAGACGGTAGTCAACGTGTTACAGACTT GATG-3’; 5’-GATGTCAAGCAGTCCTAACTTTGAGGCAGAGTCCGTGACGCTCAGT ATCG-3’; 5’-CATCAAGTCTGTAACACGTTGACTACCGTC-3’) and the 5’-overhang partial duplex DNA (5’-^32^P-labeled 5’-GCAGTGGCTATCGTATAGTATTAGGTTGGTGACCC CGTAAGGAAAGTTTT-3’; 5’-AAAACTTTCCTTACGGGGTCACCAA CCTAATA-3’). DNA cleavage was monitored as previously described ^7^. DNA cleavage assay was carried out using the previously described procedure with modification ^19^. Briefly, reaction mixtures (20 μl) containing 50 mM Tris-HCl (pH 7.5), 1 mM DTT, 5% glycerol, BSA (2 μg), and indicated amount of MgCl_2_ and salt were incubated with indicated amounts of wt-Metnase or the mutant in the presence of 60 fmol of radiolabeled DNA. After incubation at 37°C for indicated amount of time, reaction mixtures were analyzed by 12% polyacrylamide gel electrophoresis containing 8 M urea for DNA cleavage. The cleavage product was quantified using PhosphorImager and ImageQuant software (Molecular Dynamics).

### Interaction of Metnase with DNA using DNA pulldown assay

The 3’-biotinylated 5’-flap DNA (50 pmol) was incubated with streptavidin agarose pre-treated with BSA, and rotated for 30 min at RT in the presence of 1.0 ml of a buffer (50 mM Tris-HCl, pH 7.5, 25 mM NaCl, 1.0 mM DTT, 0.1 mg/ml of BSA). After adding 1.0 μg of wt-Metnase to the DNA-Streptavidin agarose, the mixtures were rotated for 30 min at room temperature and centrifuged for 5 min at 5,000 rpm. The precipitates were collected and washed once with 1.0 ml of the washing buffer (50 mM Tris-HCl, pH 7.5) containing various NaCl concentrations. After rotation for 5 min, the pellets were centrifuged for 5min at 5,000 rpm at room temperature and analyzed by Western blot.

### Colocalization of RPA and γ-H2AX

Cells (2.0 x 10^5^ cells/well) were grown on poly D-Lysine coated coverslips (neuVitro, 18 mm dia.), and 48 hrs later were treated with 2 mM HU for 3 hrs, washed three times with growth media, and incubated in fresh media for the indicated time. After briefly washing cells with PBS, cells were fixed for 5 min in ice-cold methanol at -20°C. The slips were then blocked for 30 min with 5% BSA in PBS, and incubated with a mouse anti γ-H2AX monoclonal antibody (Millipore, 05–636) for 1.5 hr at room temperature. The slides were washed four times with 0.2% Tween-20, and incubated for 1 h at room temperature with a donkey anti-mouse Alexa Fluor 488 secondary antibody (Invitrogen) in a wet chamber covered by aluminum foil. After washing 4 times with 0.2% Tween-20 PBS, samples were mounted (VECTASHIELD® Mounting Medium with DAPI, VECTOR Laboratories), and nuclei were visualized using a confocal microscope (Zeiss LSM 510 NLO, 63X oil immersion objective).

### DNA fiber analysis after dual labeling of chromosomal replication with CldU and IdU

DNA fiber analysis was carried out as described previously [5]. Briefly, cells grown in 6-well dishes (6 x 10^5^ per well), 20 μM IdU was added to growth medium and incubated for 20 min at 37°C. After washing with fresh medium, cells were treated with 5 mM HU for 60 min or mock treated. Medium was replaced with fresh medium containing 100 μM CldU, cells were further incubated for indicated times at 37°C. Cells were harvested, resuspended in PBS, and ~1000 cells were transferred to a positively charged microscope slide (Superfrost/Plus, Daigger), and processed for DNA fiber analysis as previously described [5]. Slides were mounted in PermaFluor aqueous, self-sealing mounting medium (Thermo Scientific) and DNA fibers were visualized using a confocal microscope (Olympus, FV1000D, 63X oil immersion objective). Images were analyzed using the OLYMPUS FLUOVIEW software.

## Results

### The Metnase SET domain is essential for recovery from DNA damage at the replication forks following HU treatment

**Fig 1:**
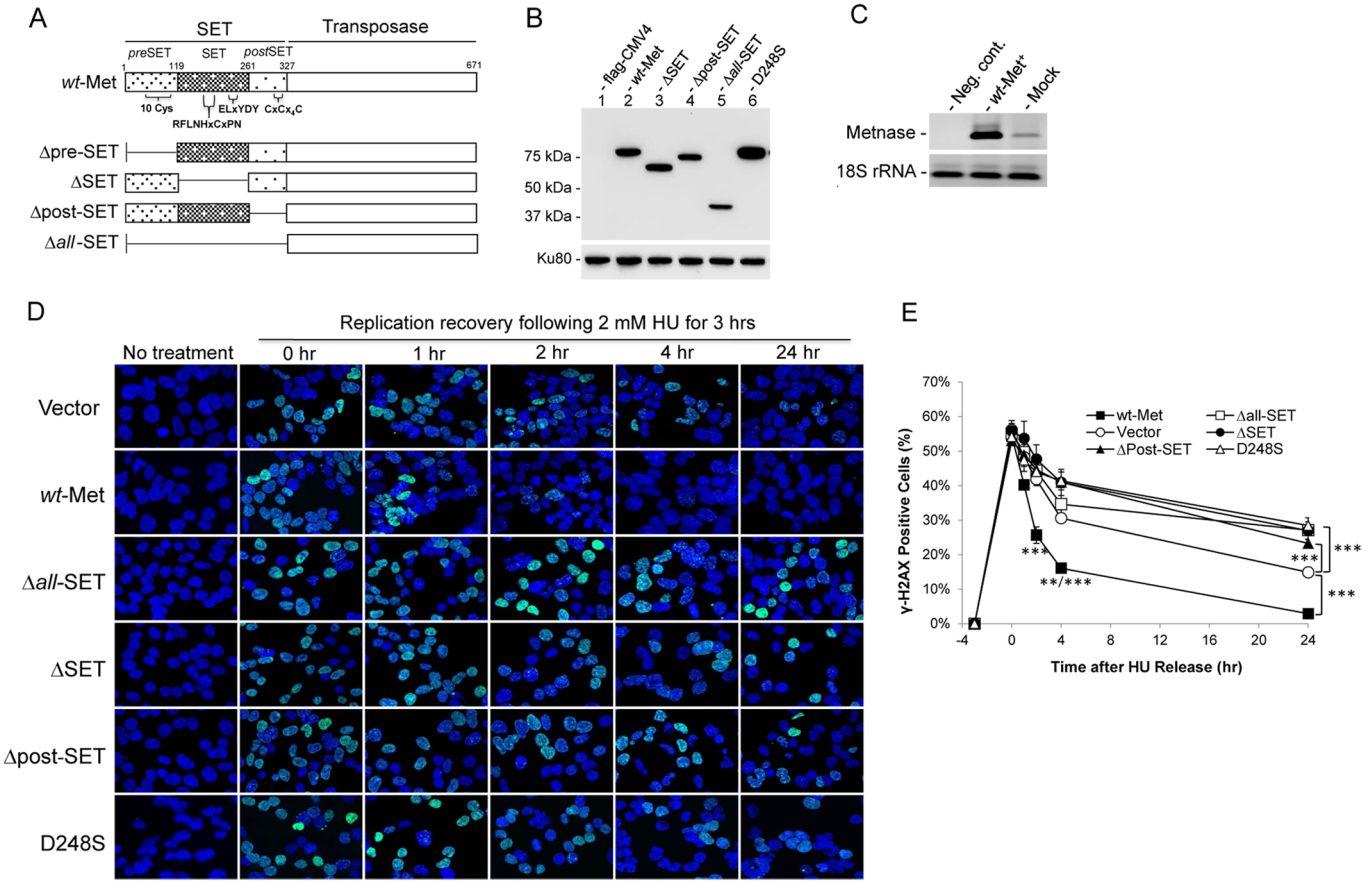
The Metnase SET domain is necessary for replication recovery after long exposure to hydroxyurea (HU). (A) Schematic diagram of Metnase. The Metnase SET domain comprises pre-SET (aa 14–118), SET (aa 119–260), and post-SET (aa 261–326) domains. The *pre* SET domain contains a cysteine-rich putative Zn^+2^ binding motif (10 Cys), while the SET domain has the SAM binding motif (207-RFLNHxCxPN----ELxYDY-248) for histone lysine methyltransferase activity. The *post*-SET domain also contains a cysteine-rich putative Zn^+2^ binding motif (CxCx_4_C). (B) Western blot analysis of flag-tagged wt-Metnase and the SET mutants that were stably expressed in HEK293 cells. Thirty μg of cell extracts were loaded onto 10% SDS-PAGE for immunoblot analysis. Ku80 was used as a loading control. (C) Metnase expression in control HEK293 cells (mock) and wt-Metnase overexpressor (wt-Met^+^) was analyzed by RT-PCR. (D) Representative confocal microscope images of HEK293 cells stably transfected with pCMV4 vector (top row), wt-Metnase (second row), and the SET mutants (third to sixth row) following HU treatment. After treatment of 2 mM HU for 3 hrs, cells were released into fresh media at indicated times, stained with DAPI (blue) and an antibody to γ-H2AX (green), and imaged by confocal microscopy. (E) Quantitation of γ-H2AX-positive cells in panel D. Plots were average percentages (±SD) of γ-H2AX-positive cells. An average of 200 cells were counted per slide, 6 slides per experiment. **, P<0.01; ***, P<0.005, t tests.

Metnase (also known as SETMAR) is a SET-transposase chimeric protein in human that promotes DSB repair and restart of stalled replication forks [1, 2, 4–6, 13]. While the SET domain with its H3K36me2 activity plays a unique role in NHEJ repair [3], its role in restart of stalled replication fork is unclear. To investigate a potential role for the SET domain in replication restart, we generated several deletion mutants lacking pre-SET (aa 14–118), SET (aa 120–256), post-SET (aa 273–302), and the entire SET (*all*-SET) domains (Fig 1A). These SET deletion mutants, except Δpre–SET, were stably expressed in HEK293 cells (Fig 1B) and their expression levels were approximately 3–8 times higher than the endogenous Metnase (Fig 1C).

**Fig 2:**
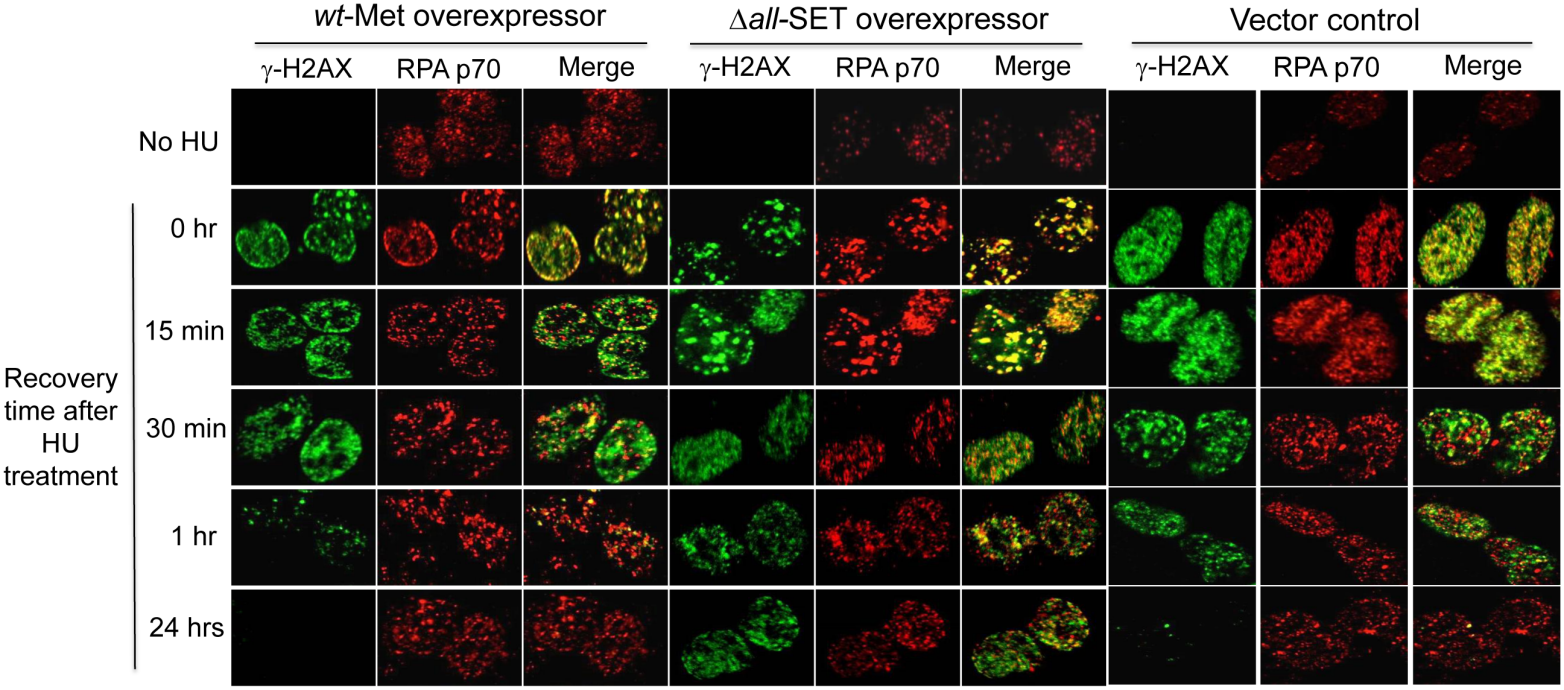
The Metnase SET domain is necessary for Metnase function in damage recovery prior to replication restart following HU treatment. Representative images of γ-H2AX and RPA p70 foci in HEK293 cells stably transfected with pCMV4 vector, wt-Metnase, and the SET deletion mutant (Δ*all*-SET), as indicated on the top. After treatment with 2 mM HU for 3 hrs, cells were released into fresh media at indicated times, stained with DAPI (blue) and antibodies to γ-H2AX (green) and RPA p70 (red), and imaged by confocal microscopy.

To see an impact of the Metnase SET domain on cell sensitivity, HEK293 cells expressing SET deletion mutant were compared with wt-Metnase for recovery from DNA damage at the replication forks by measuring the disappearance of γ-H2AX foci following treatment of cells with 2 mM HU for 3 hrs. HEK293 cells harboring endogenous wt-Metnase were used to determine the role of Metnase-mediated DNA cleavage activity in replication fork recovery [5] therefore we used the same cells to comparatively analyze the SET deletion for Metnase function. HU-induced γ-H2AX foci in wt-Metnase overexpressor (wt-Met) mostly disappeared in 2 hrs after the release, while cells overexpressing the SET deletion mutant very slowly responded (Fig 1D and 1E). Cells overexpressing a substitution mutant lacking HLMT activity (D248S) [3] also showed a slow replication recovery after HU release. We next tested cells expressing the SET deletion mutant compared with wt-Metnase for fork recovery from DNA damage after HU release. For this, HEK293 cells overexpressing either wt-Metnase or the SET deletion mutant (Δ*all* -SET) were examined for γ-H2AX foci and the large subunit of replication protein A (RPA70) after release from HU treatment (Fig 2). Gamma-H2AX foci represent an early event of DNA damage response at stalled replication fork, while RPA70 foci are formed at either blocked or active replication forks [30]. In a merged image, RPAs are colocalized with γ-H2AX at stalled replication forks, while in active replication forks RPAs are separately localized from γ-H2AX. RPA and γ-H2AX were colocalized immediately after HU release in both wt-Metnase (wt-Met) and the mutant overexpressor (Δ*all*-SET) (Fig 2) [31]. A portion of RPA foci in wt-Metnase overexpressor were separately localized from γ-H2AX foci 15 min after HU release, whereas RPA were mostly colocalized with γ-H2AX in cells overexpressing Δ*all*-SET (Fig 2). Most RPA foci in cells overexpressing wt-Metnase were localized at active replication forks an hour after HU release, whereas a significant number of RPA foci in cells overexpressing Δ*all*-SET remained at stalled replication forks (Fig 2). Together, these results suggest that the Metnase SET domain has a unique role in recovery from DNA damage at the replication forks following HU release.

### The SET domain is necessary for accelerating the restart of stalled replication forks

**Fig 3:**
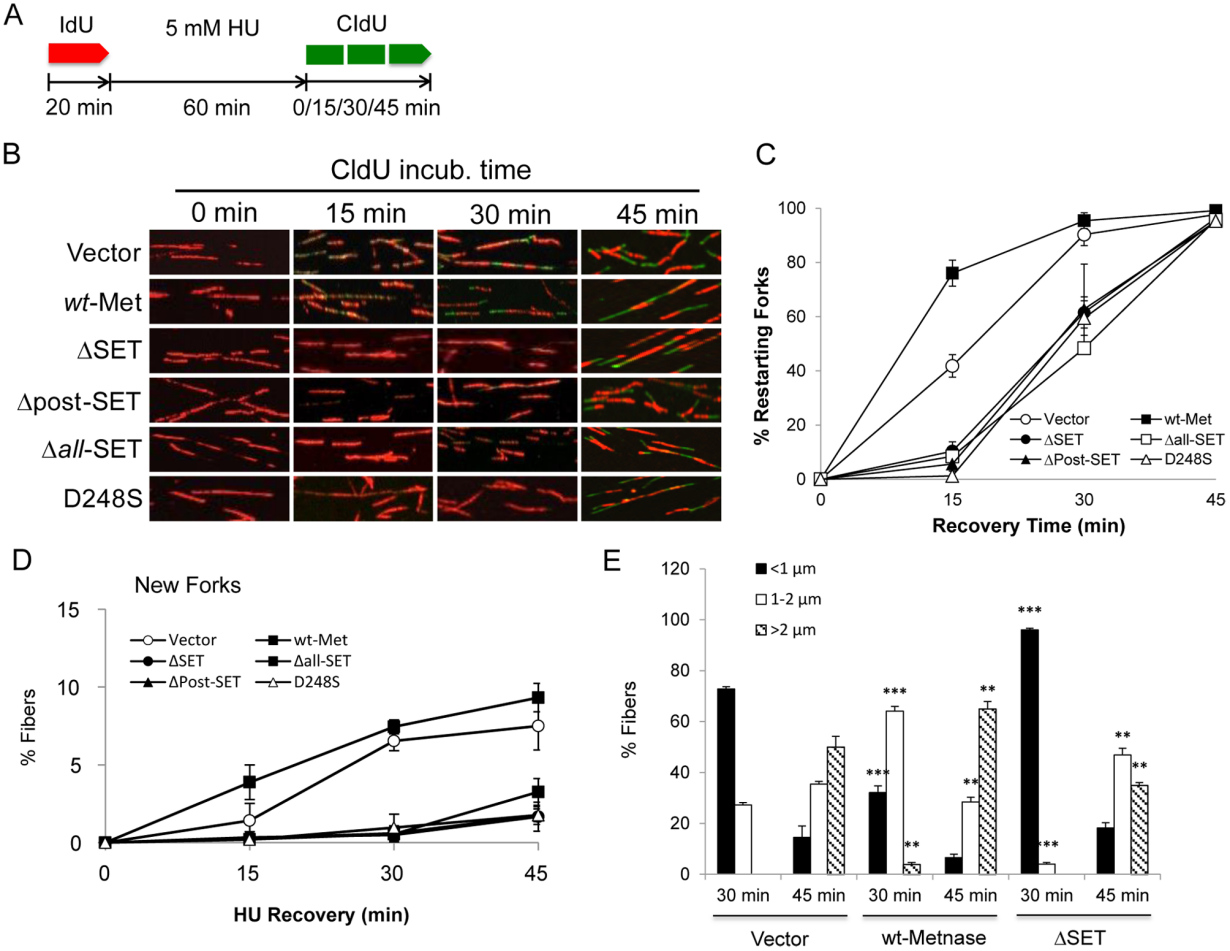
The Metnase SET domain has a crucial role in replication fork restart following HU treatment. (A) Dual labeling protocol for replication restart using DNA fiber analysis. Cells were pulse-labeled with IdU (red) for 20 min, treated with 5 mM HU for 60 min, and labeled with CldU (green) for 0, 15, 30, and 45 min. (B) Representative confocal microscope images of replication tracks from HEK293 cells stably expressing vector (top row), wt-Metnase (2^nd^ row), and the SET deletion/substitution mutants (3^rd^- 6^th^ rows). Cells were treated with 5 mM HU prior to pulse labeling with CldU for indicated amounts of time. (C-D) Average percentage (±SD) of restarted forks (red plus green; panel C) and new forks (green only; panel D) for three independent experiments in which 150–200 fibers were scored per each determination. (E) Fiber lengths were measured by using Olympus FV1—ASW 3.0 software. Average lengths of 150–200 fibers were scored in triplicates per each determination. **, P<0.01; ***, P<0.005 (compared with vector control), t tests.

To further understand the SET domain’s role(s) in replication restart, we carried out DNA fiber analyses with cells overexpressing the SET deletion mutants compared with wt-Metnase. Cells were incubated with IdU for 20 min prior to HU treatment and released into fresh media in the presence of CldU for various times (Fig 3A). All of fibers had only red signals immediately after HU release into media containing CldU (0 min CldU; Fig 3B). Control and wt-Metnase overexpressor started to show adjacent red-green signals in 15 min CldU indicative of restarted forks. On the other hand, cells overexpressing the SET deletion mutants (ΔSET, Δpost-SET, and Δ*all*-SET) exhibited a delay in restart after HU release (Fig 3B). Quantitative analysis showed that a significant delay in fork restart with cells overexpressing the SET deletion mutant, but the level of restarting forks (%) in the SET mutants reached to the wt-Metnase in 45 min after HU release (Fig 3C). Interestingly, a substitution mutant (D248S) defective in HLMT activity [3, 6] also showed a delayed fork restart (Fig 3B), suggesting that Metnase-mediated HLMT activity is involved in fork restart as well as in DSB repair [3].

### The SET domain is essential for the 5’ ss-overhang cleavage of fork DNA structures

**Fig 4:**
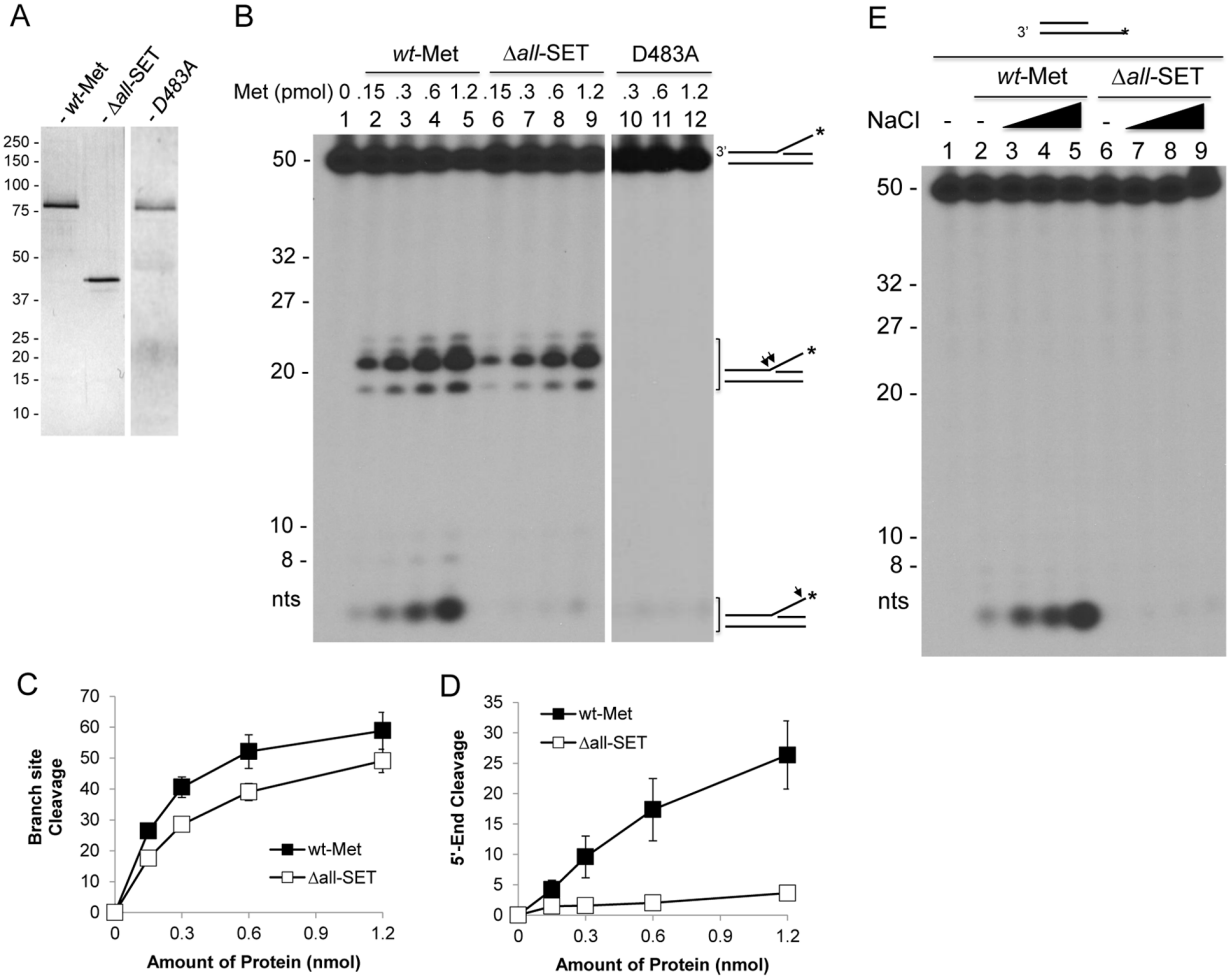
The Metnase SET domain is necessary for the 5’-end cleavage of ss-overhang DNA. (A) Silver staining of purified wt-Metnase (*wt-*MET), the SET deletion mutant (Δ*all*-SET), and a nuclease-dead mutant (D483A) following 10% SDS-PAGE. (B) The SET domain is essential for cleavage of the 5’-flap DNA. Reaction mixtures (20 μl) containing the 5’-^32^P-labeled flap DNA (60 fmol) and increasing amounts of *wt*-Metnase or the mutant (Δ*all*-SET or D483A) were incubated at 37°C in the presence of 2 mM MgCl_2_ for 90 min, and cleavage products were analyzed by 12% PAGE containing 8M urea. Numbers on the left indicated DNA size makers. (C-D) Cleavage of the branch site (panel C) and the 5’ end of ss-overhang (panel D) of a 5’-flap DNA shown in Panel B was quantified using a PhosphorImager and ImageQuant software (Molecular Dynamics). (E) Cleavage of the 5’ end of the 5’-^32^P-labeled partial duplex DNA with wt-Metnase and the SET deletion mutant. Indicated amount of wt-Metnase or the Δ*all*-SET was incubated with the 5’-^32^P-labeled (*) 5’-ss-overhang DNA (60 fmol) for 90 min at 37°C prior to 12% PAGE analysis (+ 8M urea). Arrows on the right mark the cleavage sites on the 5’-^32^P-labeled (*) DNA.

A full recovery of delayed fork restart in 45 min with the SET deletion mutant (Fig 3C) was also observed with cells overexpressing a nuclease-dead mutant (D483A) [5], suggesting that the SET domain may be involved in Metnase-mediated DNA cleavage activity. To test this possibility, the SET deletion mutant was purified from HEK293 cells overexpressing Δ*all*-SET (Fig 4A) and examined their DNA cleavage activity. As shown previously [5], wt-Metnase mediated cleavage at the branch site and the 5’ end of ss-overhang with a 5’-flap DNA, while a nuclease-dead mutant (D483A) showed no cleavage activity (Fig 4B). The SET deletion mutant (Δ*all*-SET) mediated cleavage at the branch site but not the 5’-end of a 5’-flap DNA (Fig 4B). The Δ*all*-SET also failed to show cleavage at the 5’ end of ss-overhang with a partial duplex DNA under the conditions where wt-Metnase effectively did (Fig 4C), suggesting that the SET domain is necessary for Metnase-mediated cleavage at the 5’ end of ss-overhang with fork and non-fork DNA.

**Fig 5:**
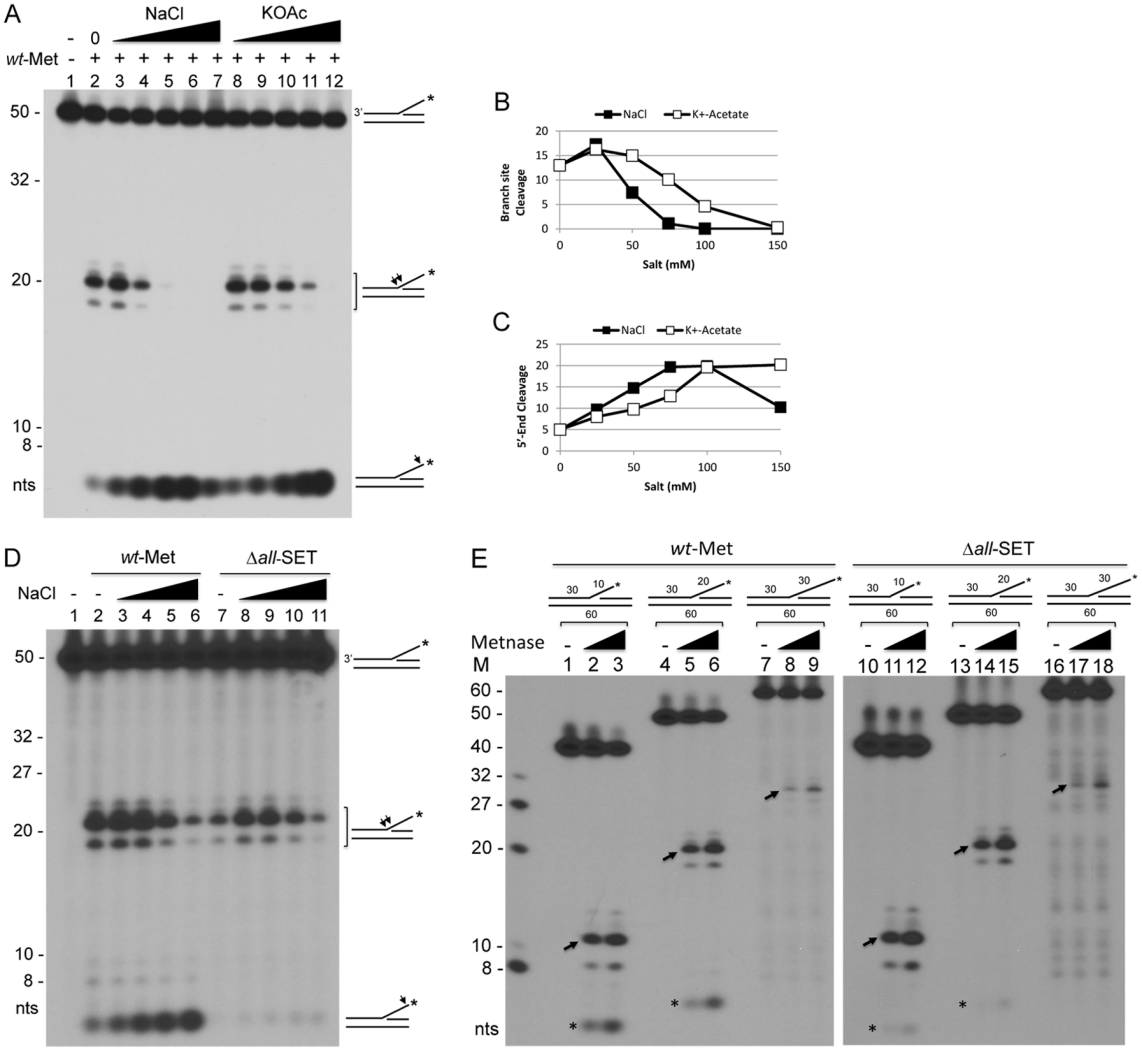
The Metnase SET domain is required for a preferential cleavage of the 5’ end of a 5’-flap DNA in the presence of high salt. (A) Metnase prefers cleavage of the branch site at low salt, while it likes cleavage of the 5’-end of a 5’-flap DNA in the presence of high salt. *Wt*-Metnase (50 ng) was incubated with 60 fmol of the 5’-^32^P-labeled (*) DNA for 120 min in the presence of increasing concentrations (0, 25, 50, 75, 100, and 150 mM) of NaCl or potassium acetate (KOAc) prior to 12% denatured PAGE (+ 8M urea) analysis. Arrows on the right mark the cleavage sites on the ^32^P-labeled (*) 5’-flap DNA. (B-C) Metnase-mediated cleavage of the branch site (panel B) and the 5’ end of ss-overhang (panel C) of a 5’-flap DNA shown in Panel A was quantified. (D) Cleavage of the 5’ end of a 5’-^32^P-flap DNA with wt-Metnase and the SET deletion mutant in the presence of varying salt concentrations. Fifty ng of wt-Metnase or the Δ*all*-SET was incubated with a 5’-^32^P-flap DNA in the presence of increasing concentrations (0, 25, 50, 75, and 100 mM) of NaCl. Arrows on the right mark the cleavage sites on the 5’-^32^P-labeled (*) flap DNA. (E) Metnase exhibits branch site cleavage of the 5’-flap DNA with different sizes of ss-overhang. Wt-Metnase (50 or 100 ng; lanes 1–9) or the SET deletion mutant (lanes 10–18) was incubated with the 5’-^32^P-labeled 5’-flap DNA in different length of ss-overhang (10-mer, lanes 1–3 & 10–12; 20-mer, lanes 4–6 & 13–15; 30-mer, lanes 7–9 & 16–18). Arrows mark the major branch site cleavage with the ^32^P-labeled 5’-flap DNA. M on the left represents DNA markers.

Since the SET domain is crucial for Metnase-mediated cleavage at the 5’ end but not at the branch site of fork DNA, we questioned whether cleavage of these two sites actually occurs separately. To test that, we carried out Metnase-mediated cleavage of a 5’-flap DNA in the presence of varying salt levels. Cleavage at the branch site of a 5’-flap DNA peaked at 25 mM NaCl but sharply declined with higher salt concentrations (Fig 5A and 5B), while cleavage at the 5’-end of ss-overhang was elevated with increasing salt levels (Fig 5A and 5C). This observation suggests that Metnase prefers the branch site cleavage of a 5’-flap DNA in the presence of low salt, while it favors cleavage at the 5’-end of ss-overhang with higher salt. We then examined the SET deletion mutant (Δ*all*-SET) for cleavage of a 5’-flap DNA substrate in the presence of increasing salt levels. Similar to wt-Metnase, Δ*all*-SET mediated cleavage at the branch site of a 5’-flap DNA peaked at 25 mM NaCl, but it showed little or no cleavage at the 5’ end of ss-overhang regardless of salt concentration (Fig 5D). To further understand the SET domain’s role in DNA cleavage, we examined cleavage activity of wt-Metnase and the SET deletion mutant in the presence of different sizes of 5’-flap DNA. Cleavage sites on the branch and the 5’ end of flap DNA varied with different sizes of flap DNA (Fig 5E), while the SET deletion only affects cleavage of the 5’ end and not the branch site. Together, these observations suggest that the SET domain has a positive role in Metnase-mediated cleavage of fork DNA, which may be directly linked to its role in restart of replication.

### HLMT activity is not involved in Metnase-mediated cleavage of the 5’ ss-overhang DNA

**Fig 6:**
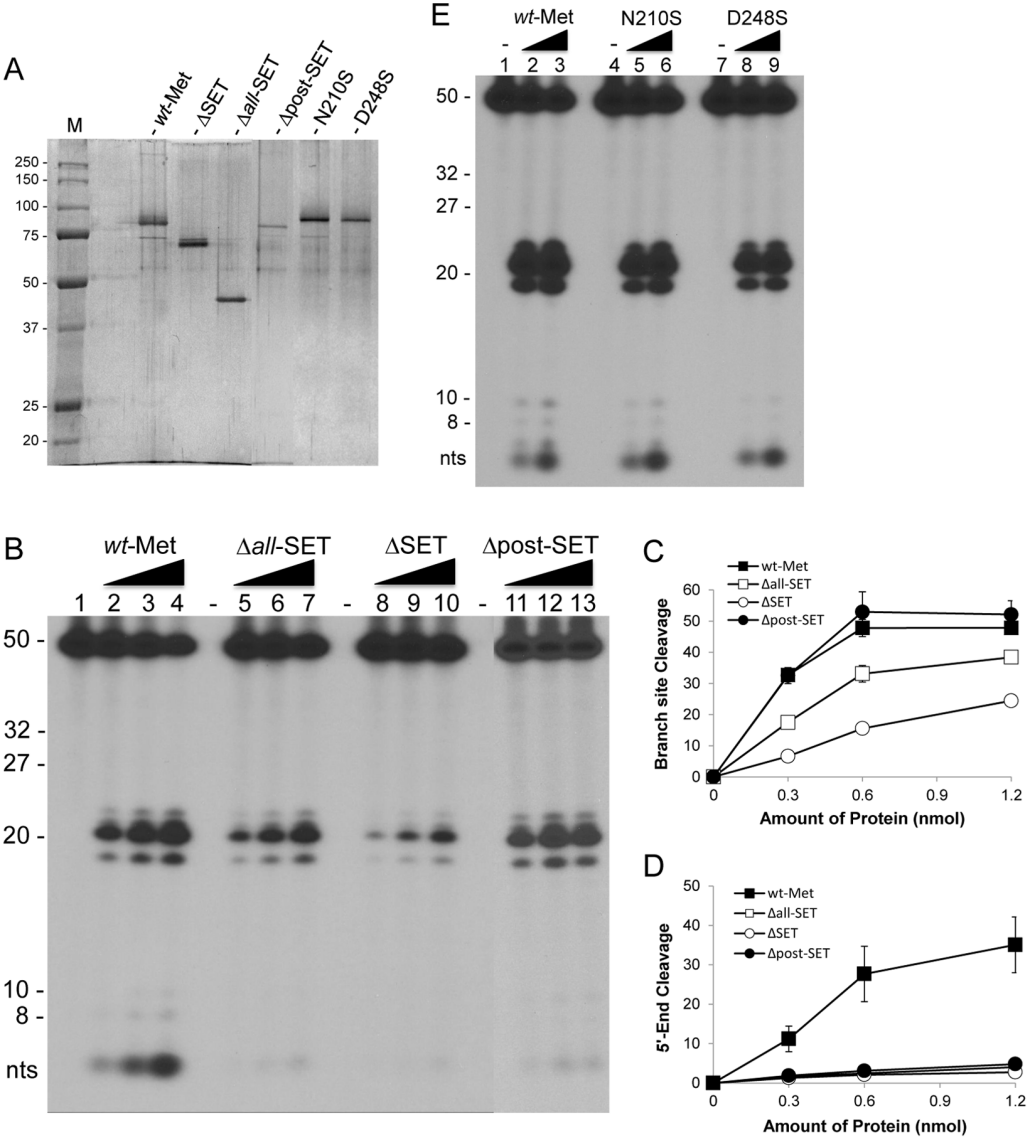
The Metnase SET domain but not its HLMT activity is essential for cleavage of the 5’ end of a 5’-flap DNA. (A) Silver staining of purified wt-Metnase (*wt-*MET) and the SET deletion and the substitution mutants following 10% SDS-PAGE. (B) Cleavage of the 5’ end of a 5’-flap DNA with the SET deletion and the substitution mutants. Increasing amounts of *wt*-Metnase and the SET domain deletion mutant (panel B) were incubated with 60 fmol of a 5’-^32^P-flap DNA for 120 min prior to 12% denatured PAGE (+ 8 M urea) analysis. (C-D) Metnase-mediated cleavage of the branch site (panel C) and the 5’ end of ss-overhang (panel D) of a 5’-flap DNA shown in Panel B was quantified. (E) Increasing amounts of *wt*-Metnase and the substitution mutants lacking HLMT activity (N210S & D248S) were examined for cleavage of the 5’-flap DNA.

To further understand the SET domain’s role in fork DNA cleavage, we prepared several SET deletion mutants (Δ*all*-SET, ΔSET, and Δpost-SET; Fig 6A) for cleavage at the 5’ end of ss-overhang of a 5’-flap DNA. All the SET deletion mutants we tested mediated branch site cleavage but showed little or no 5’ end of ss-overhang cleavage (Fig 6B). The Metnase SET domain is not only responsible for dimethylation of H3K36 [3, 6] but also crucial for automethylation at K485 within the catalytic pocket [8]. The latter could affect Metnase-mediated cleavage at the 5’ end of ss-overhang DNA. We therefore examined two substitution mutants (N210A and D248S) lacking HLMT activity [3, 6] compared with wt-Metnase for DNA cleavage activity. Similar to wt-Metnase, both mutants showed cleavage at both branch site and the 5’ end of ss-overhang with a 5’-flap DNA (Fig 6C), suggesting that automethylation status of Metnase may not affect fork DNA cleavage activity.

### The SET domain is not involved in the Metnase-DNA interaction

**Fig 7:**
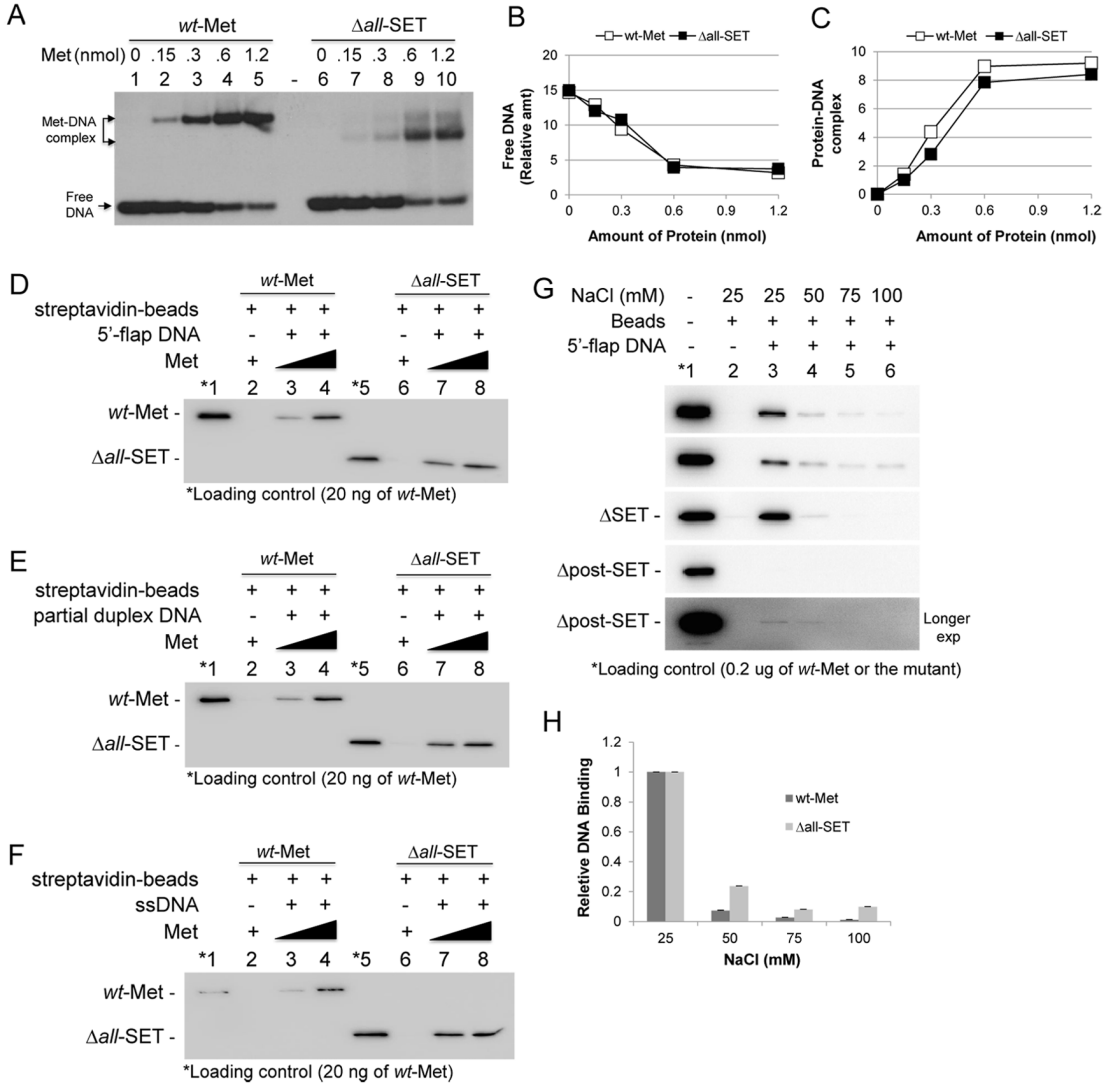
The Metnase SET domain is not involved in the Metnase-DNA interaction. **(A)** Indicated amounts of wt-Metnase or the Δ*all*-SET mutant were incubated with 400 fmol of 5’-^32^P-labeled TIR DNA. Following 15 min incubation at 25°C, the protein–DNA complexes were analyzed by 5% native PAGE in the presence of 1X TBE. (B-C) Quantitation of free DNA and the Metnase-TIR complex. For quantitation, individual bands were excised from dried gel and measured for radioactivity. (D-F) Interaction of wt-Metnase and the SET deletion mutant (Δ*all*-SET) with DNA using Streptavidine pulldown assay. Flag-tagged *wt*-Metnase or Δ*all*-SET protein (1.0 and 2.0 μg) was incubated with 50 pmol of the 3’-biotinylated 5’-flap DNA (panel D), a partial duplex DNA (panel E), or ssDNA (panel F) for protein-DNA binding by Streptavidin-agarose beads (see Experimental Procedures for the details). The protein-DNA interaction was analyzed by Western blot using an anti-flag antibody. (G) Salt sensitivity of wt-Metnase and the SET deletion mutants in their interaction with a 5’-flap DNA. Wt-Metnase or the SET deletion mutant (2.0 μg) was incubated with 50 pmol of the 3’-biotinylated 5’-flap DNA in the presence of varying concentrations of NaCl prior to DNA pull-down with streptavidin-agarose beads. The protein-DNA interaction was analyzed by Western blot using an anti-flag antibody. (H) The protein-DNA complexes (panel G) were quantified by Molecular Imager ChemiDoc XRS using Quantity One^**®**^ analysis software program (BioRad).

Differential salt sensitivity in Metnase-mediated cleavage at the branch site and the 5’ end of ss-overhang DNA (Fig 5) may be due a change in Metnase-DNA interaction under different ionic strength. For example, Metnase may bind to the branch site of a 5’-flap DNA at low ionic strength, while the 5′-terminus could be a preferred site for Metnase loading onto a 5′-flap DNA for the 5’ ss-overhang cleavage with higher salt concentrations. In fact, a motif within the SET protein was previously shown to be involved in ssDNA binding [32]. We therefore examined the SET deletion mutant compared with wt-Metnase for interaction with DNA in the presence of various salt concentrations. Similar to wt-Metnase [13, 16, 17, 20, 33], the SET deletion mutant (Δ*all*-SET) showed a stable interaction with the 5’-terminal inverted repeat (TIR) DNA (Fig 7A–7C), indicating that the SET domain is not involved in the Metnase-TIR interaction. We next tested whether the SET domain is involved in binding to non-TIR fork and non-fork DNA. For this, wt-Metnase and the SET deletion mutant were incubated with the 3’-biotin-labeled 5’-flap DNA, and the protein-DNA interaction was scored by Western blot following a pull-down with streptavidin-agarose beads. Both wt-Metnase and the SET deletion mutant were effectively pulled down with a 5’-flap DNA (Fig 7D), a partial duplex DNA (Fig 7E) or a ssDNA(Fig 7F), suggesting that the SET domain is not involved in the Metnase-DNA interaction. We further tested the SET deletion mutants for binding to a 5’-flap DNA in the presence of increasing salt concentrations. The Metnase-DNA interaction was observed with low salt (25 mM NaCl) but was sharply declined in the presence of higher salts (Fig 7G and 7H). With an exception of Δpost-SET mutant that showed an extremely low DNA binding activity, all other SET deletion mutants exhibited DNA binding activity similar to wt-Metnase (Fig 7G). Given that the post-SET domain (aa 261–327) is adjacent to the helix-turn-helix (HTH) DNA binding domain motif (aa 334–381), deletion of the post-SET domain may negatively impact its DNA binding activity. Salt sensitivity of the Metnase-DNA interaction correlated well with Metnase-mediated branch site cleavage activity (Fig 5), suggesting that Metnase binding to a 5′-flap DNA occurs in favor of the branch site cleavage at low salt, while the 5′-terminus could be a preferred site for Metnase loading onto a 5′-flap DNA in the presence of higher salts, resulting in the 5’ ss-overhang cleavage with fork and non-fork DNA.

## Discussions

Metnase is a SET-transposase chimeric protein in humans that promotes DNA repair and restart of replication forks. The SET domain is responsible for dimethylation of H3K36, a histone mark associated with ‘open’ chromatin and improved association of early DNA repair components such as NBS1 and Ku70 at DSB sites for NHEJ repair, but its involvement in replication restart is not known. The work presented here demonstrates that the Metnase SET domain is essential for accelerating the restart of stalled replication fork and the 5’ ss-overhang cleavage of fork DNA.

The SET domain of Metnase has two conserved motifs, RFLNHxCxPN and ELxYDY that are responsible for the interaction with SAM and dimethylation of H3K36 [3, 6]. The substitution mutation at these sites (N210A and D248S) abolished DSB repair and interaction with topoisomerase IIα, respectively [3, 6, 8], but also caused a delay in fork restart (Fig 3B and 3C), suggesting that Metnase-mediated HLMT activity plays a role in accelerating stalled replication fork. Given that H3K36me2 is necessary for an improved association of DNA repair components for NHEJ repair [3], H3K36me2 may also increase accessibility of damage signaling or checkpoint factors and repair factors to stalled replication fork [29]. A mutation at the catalytic motif (DDN) destroyed Metnase function in replication restart, indicating that Metnase-mediated DNA cleavage activity plays a unique role in replication restart [5]. The transposase domain possesses DNA binding and the catalytic motifs that account for Metnase’s endonuclease action [1, 5, 13, 20], but the present study demonstrated that the Metnase SET domain is also essential for cleavage at the 5’ end of ss-overhang with fork and non-fork DNA. A mutant defective in HLMT activity failed to support accelerated restart of replication forks (Fig 3B) but retained the 5’ end cleavage activity (Fig 6C), indicating that the SET domain plays a role in replication restart via its functions in HLMT and the 5’ end cleavage of ss-overhang with fork DNA. Another mechanism by which Metnase could promote fork restart is through its interactions with replisome factors including PCNA and RAD9. Metnase may interact with these proteins via a conserved non-canonical PIP-Box (190-xxxMExfv-197) within the SET domain. Given the well-established role of RAD9 in the intra-S checkpoint response [34], Metnase could promote fork restart by influencing checkpoint activation or downstream checkpoint-dependent processes such as inhibition of origin firing.

Metnase mediates branch site cleavage of a 5’-flap DNA in the presence of a low salt while it prefers the cleavage of the 5’ end of ss-overhang DNA with higher salt concentrations (Fig 5). A change in cleavage site(s) under different salt concentrations may be due to its binding preference to DNA substrate. For example, Metnase binding to a 5’-flap DNA occurs in favor of branch site cleavage in the presence of low salt, while the 5′ terminus might be a preferred site for Metnase loading onto a 5′-flap DNA in the presence of higher salt. This notion is well supported by a competition experiment where the branch site cleavage was significantly lowered by addition of unlabeled 5’-flap DNA in the presence of low salt, but was not affected by non-labeled partial duplex DNA (S1 Fig). What role does the SET domain play in the 5’ end cleavage of ss-overhang DNA? Cleavage of the 5’ end of ss-overhang DNA is more closely resembles flap endonuclease 1 (FEN1) that cuts close to flap junctions of fork structures following recognition of the displaced flap [35] and is required for efficient restart of stalled replication forks [36]. Our Metnase-mediated DNA cleavage analysis with 5’-biotin-labeled (3’-^32^P-labeled) flap DNA indicated that the branch site cleavage was not affected by a 5’ blocking with biotin (S2 Fig), suggesting that cleavage of the branch site does not occur via the tracking model requiring free 5’ ssDNA end [35]. Given that cleavage sites on the branch and the 5’ end of flap DNA varied with different sizes of flap DNA (Fig 5E), Metnase may recognize fork DNA prior to its cleavage action. Although the SET domain is not involved in Metnase-DNA interaction, it may structurally stabilize Metnase when it carries out its cleavage action. For example, the pre-SET domain contains ten cysteine residues (62-C-x-C-x_4_-C-x_4_-C-x-C-x_11_-C-x_16_-C-x_3_-C-x-C-x_3_-C-115) that likely coordinate zinc ions to form a triangular cluster [37] (Fig 1A), a structural component necessary for stabilizing the SET and the transposase domains for DNA cleavage action. The post-SET domain has four cysteines (274-C-x-C-x_4_-C-x_11_-C-293) that also likely form a zinc cluster (Fig 1A) and stabilizes the catalytic action [37], while it brings in the C-terminal residues for S-adenosylmethine-binding and histone tail interactions necessary for HLMT activity [37]. Structural stabilization of the SET-transposase chimera may not be directly involved in DNA binding (Fig 7F), but it could significantly impact on the 5’ end cleavage of ss-overhang with fork and non-fork DNA (Fig 6). The 5′ terminus is a good substrate for a flap endonuclease (FEN-1) loading onto a 5′ flap DNA [38, 39] and is also a favorable entry for loading of transposase onto DNA substrate [40]. In Metnase, electrostatic repulsion by the 5’ terminal phosphate may allow increased breathing of the substrate into a pseudo-flap configuration, providing the active form of the substrate for cleavage of the 5’ end of ss-overhang. The Metnase SET domain may stabilize the catalytic motif when it directly interacts with negatively charged ssDNA extension and a 5’-flap DNA for ss-overhang cleavage [5]. Considering that a nuclease-dead mutant lowers end-joining activity via directly affecting DNA end processing [1], the SET domain may also be necessary for DNA end joining. In conclusion, the results presented here demonstrated a positive role for the SET domain in the 5’ end of ss-overhang cleavage with fork and non-fork DNA, which likely provides a new insight into the functional interaction of SET and transposase domains.

## Supporting Information

S1 Fig Effect of non-labeled competitor DNA (fork and non-fork DNA) on Metnase-mediated cleavage of branch site and the 5’ end of a 5’-flap DNA. In reaction mixtures, 50 ng of wt-Metnase (lanes 2–10) and the SET deletion mutant (lanes 12–20) was incubated with 60 fmol of the 5’-^32^P-labeled flap DNA in the presence of 4 & 8-fold excess of a 5’-flap DNA (lanes 3–4 & 13–14), partial duplex DNA (lanes 6–7 & 16–17), or ssDNA (lanes 9–10 & 19–20) for 120 min prior to 12% denatured PAGE (+ 8 M urea) analysis. The incubation was carried out in the presence of 25 mM NaCl. Arrows on the right mark the cleavage sites of the ^32^P-labeled (*) flap DNA.
(TIF)

S2 Fig Metnase-mediated cleavage of the 5’-biotin-labeled flap DNA. In reaction mixtures, increasing amount (50 and 100 ng) of wt-Metnase was incubated with 60 fmol of the 5’-biotin-labeled 3’-^32^P-labeled flap DNA for 120 min prior to 12% denatured PAGE (+ 8 M urea) analysis. The incubation was carried out in the presence of either 25 or 100 mM NaCl. Arrows on the right mark the cleavage sites of the ^32^P-labeled (*) flap DNA.
(TIF)
