# Supplementary figures and images for: The SET Domain Is Essential for Metnase Functions in Replication Restart and the 5’ End of SS-Overhang Cleavage
Source: PLoS One. 2015 Oct 5;10(10):e0139418. doi: 10.1371/journal.pone.0139418 (PMC4593633; doi:10.1371/journal.pone.0139418)

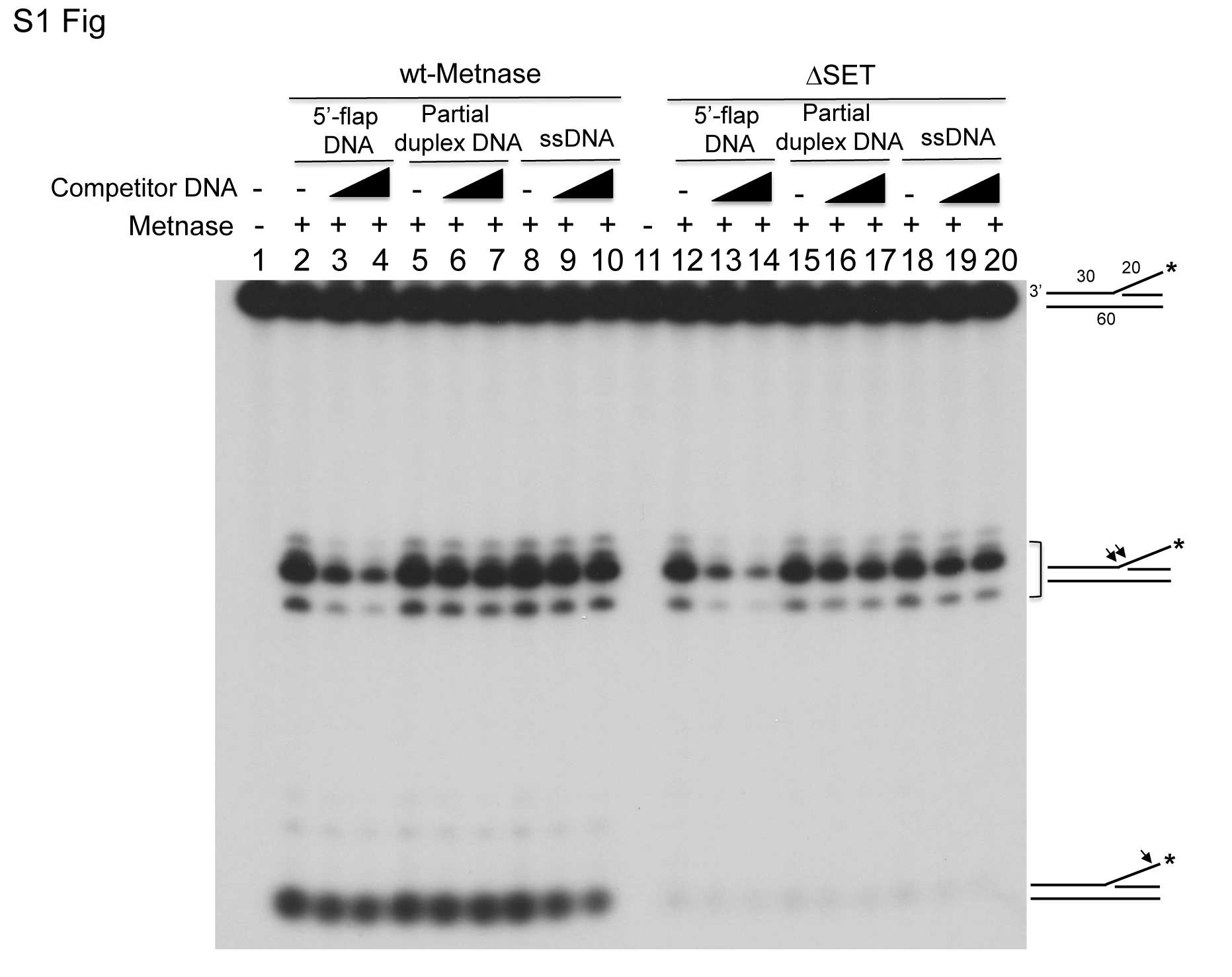

Supplement: S1 Fig — In reaction mixtures, 50 ng of wt-Metnase (lanes 2–10) and the SET deletion mutant (lanes 12–20) was incubated with 60 fmol of the 5’-32P-labeled flap DNA in the presence of 4 & 8-fold excess of a 5’-flap DNA (lanes 3–4 & 13–14), partial duplex DNA (lanes 6–7 & 16–17), or ssDNA (lanes 9–10 & 19–20) for 120 min prior to 12% denatured PAGE (+ 8 M urea) analysis. The incubation was carried out in the presence of 25 mM NaCl. Arrows on the right mark the cleavage sites of the 32P-labeled (*) flap DNA. (TIF) [file pone.0139418.s001.tif]

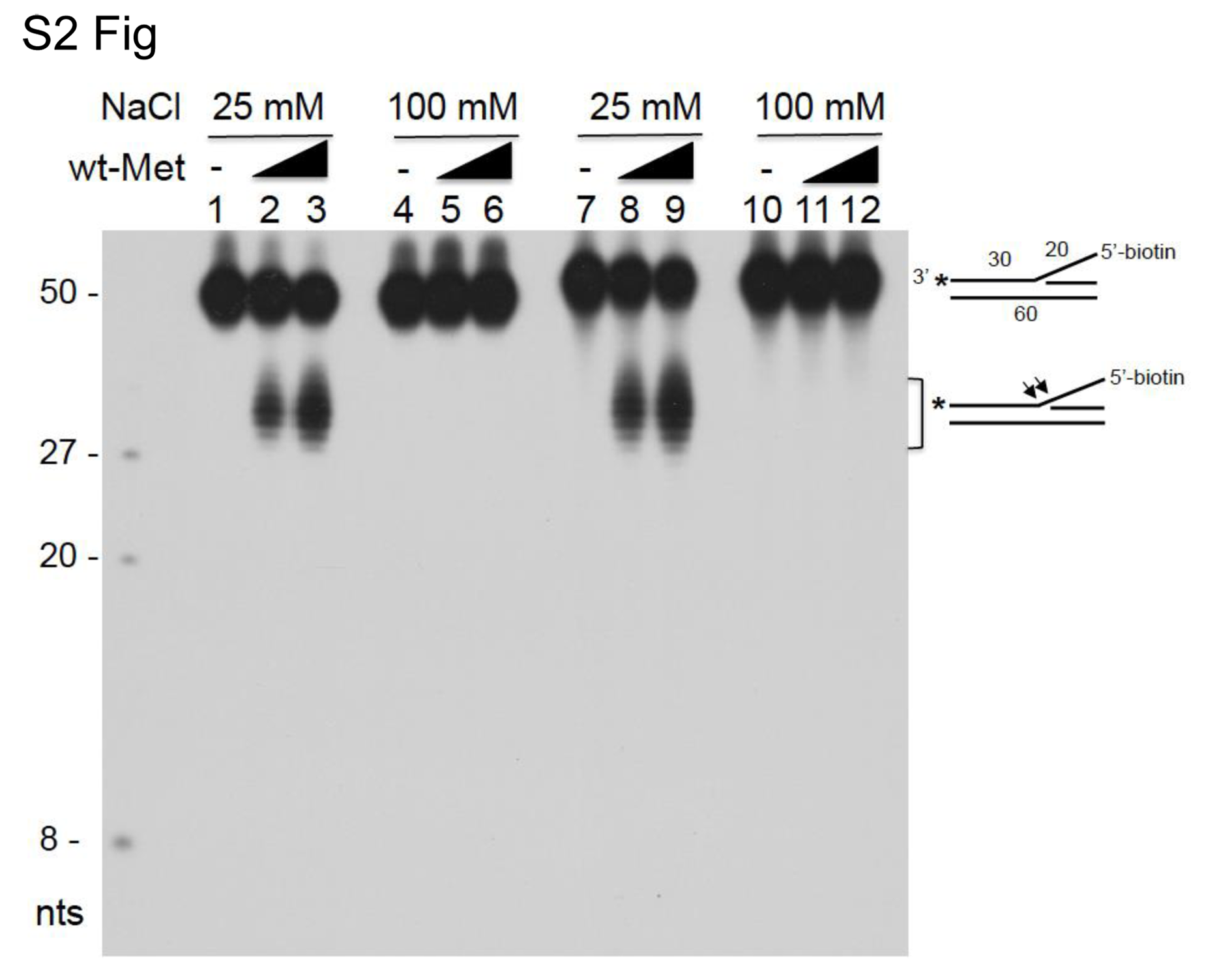

Supplement: S2 Fig — In reaction mixtures, increasing amount (50 and 100 ng) of wt-Metnase was incubated with 60 fmol of the 5’-biotin-labeled 3’-32P-labeled flap DNA for 120 min prior to 12% denatured PAGE (+ 8 M urea) analysis. The incubation was carried out in the presence of either 25 or 100 mM NaCl. Arrows on the right mark the cleavage sites of the 32P-labeled (*) flap DNA. (TIF) [file pone.0139418.s002.tif]
